# Heterogeneous Upregulation of Apamin‐Sensitive Potassium Currents in Failing Human Ventricles

**DOI:** 10.1161/JAHA.112.004713

**Published:** 2013-02-22

**Authors:** Po‐Cheng Chang, Isik Turker, John C. Lopshire, Saqib Masroor, Bich‐Lien Nguyen, Wen Tao, Michael Rubart, Peng‐Sheng Chen, Zhenhui Chen, Tomohiko Ai

**Affiliations:** 1 Division of Cardiology, Krannert Institute of Cardiology, Indianapolis, IN (P.C.C., I.T., J.C.L., P.S.C., Z.C., T.A.); 2 Florida Heart and Vascular Care, Miami, FL (S.M.); 3 Medical College of Wisconsin, Milwaukee, WI (S.M.); 4 Heart and Great Vessels Department, University of Rome La Sapienza, Rome, Italy (B.L.N.); 5 Department of Pediatrics, Wells Center for Pediatric Research, Indiana University School of Medicine, Indianapolis, IN (W.T., M.R.); 6 Chang‐Gung Memorial Hospital, Taipei, Taiwan (P.C.C.)

**Keywords:** arrhythmia, calcium, heart failure, ion channels

## Abstract

**Background:**

We previously reported that *I*_KAS_ are heterogeneously upregulated in failing rabbit ventricles and play an important role in arrhythmogenesis. This study goal is to test the hypothesis that subtype 2 of the small‐conductance Ca^2+^ activated K^+^ (SK2) channel and apamin‐sensitive K^+^ currents (*I*_KAS_) are upregulated in failing human ventricles.

**Methods and Results:**

We studied 12 native hearts from transplant recipients (heart failure [HF] group) and 11 ventricular core biopsies from patients with aortic stenosis and normal systolic function (non‐HF group). *I*_KAS_ and action potential were recorded with patch‐clamp techniques, and SK2 protein expression was studied by Western blotting. When measured at 1 μmol/L Ca^2+^ concentration, *I*_KAS_ was 4.22 (median) (25th and 75th percentiles, 2.86 and 6.96) pA/pF for the HF group (n=11) and 0.98 (0.54 and 1.72) pA/pF for the non‐HF group (n=8, *P*=0.008). *I*_KAS_ was lower in the midmyocardial cells than in the epicardial and the endocardial cells. The Ca^2+^ dependency of *I*_KAS_ in HF myocytes was shifted leftward compared to non‐HF myocytes (*K*_d_ 314 versus 605 nmol/L). Apamin (100 nmol/L) increased the action potential durations by 1.77% (−0.9% and 7.3%) in non‐HF myocytes and by 11.8% (5.7% and 13.9%) in HF myocytes (*P*=0.02). SK2 protein expression was 3‐fold higher in HF than in non‐HF.

**Conclusions:**

There is heterogeneous upregulation of *I*_KAS_ densities in failing human ventricles. The midmyocardial layer shows lower *I*_KAS_ densities than epicardial and endocardial layers of cells. Increase in both Ca^2+^ sensitivity and SK2 protein expression contributes to the *I*_KAS_ upregulation.

## Introduction

Heart failure (HF) is a major public health problem, with a prevalence of 5.8 million in the United States and over 23 million worldwide.^1^ Ventricular arrhythmia is a major cause of death in patients with HF.^2^ The mechanisms of arrhythmia in HF are attributed in part to the reduced repolarization reserve due to the upregulation of late *I*_Na_ and the downregulation of multiple major K^+^ currents (*I*_to_, *I*_Ks_, *I*_Kr_, *I*_K1_, and *I*_KATP_),^3–6^ leading to increased risk of action potential duration (APD) prolongation, early afterdepolarization (EAD), triggered activity, and ventricular arrhythmias.^7^ Although APD prolongation is proarrhythmic, acute but reversible APD shortening after ventricular fibrillation (VF)–defibrillation episodes has been observed in a rabbit model of pacing‐induced HF.^8^ The APD shortening along with persistent intracellular Ca^2+^ (Ca_i_) elevation can promote calcium transient‐triggered firing^9^ and late phase 3 EAD,^10^ leading to triggered activity and recurrent spontaneous VF. The mechanisms of acute postshock APD shortening in failing rabbit ventricles is due to VF‐induced Ca^2+^ accumulation^11^ and the upregulation of apamin‐sensitive K^+^ currents (*I*_KAS_).^12^ These studies suggest that *I*_KAS_ upregulation in failing ventricles may be antiarrhythmic by preserving the repolarization reserve but may also be proarrhythmic by acutely and excessively shortening the APD during Ca_i_ overload. However, whether *I*_KAS_ are upregulated in failing human ventricles remains unknown. The purpose of the present study is to test the hypothesis that subtype 2 of the small‐conductance Ca^2+^ activated K^+^ (SK2) channels and *I*_KAS_ are upregulated in ventricular cells from patients with HF and that *I*_KAS_ plays an important role in determining the APD in failing human ventricles.

## Methods

### Clinical Data Collection

The Human Heart Failure Tissue Collection Program is approved by the institutional review board at the Indiana University School of Medicine. The intraoperative atrial tissue collection is approved by the institutional review board of Medical College of Wisconsin and the University of Rome, La Sapienza. Written informed consents were obtained from all patients who participated in the study. Twelve native hearts from transplant recipients (HF group) and the apical tissues from 11 patients with aortic stenosis and normal systolic function who underwent apicoaortic conduit surgery (non‐HF group) were studied.^13^ Human atrial tissues were harvested during open heart surgery and preserved in liquid nitrogen. The atrial tissues were used for comparison because they are known to abundantly express SK2 proteins.^14^

### Cell Isolation

Left ventricular myocytes were isolated using enzymatic digestion methods. The heart tissues were immersed in cardioplegic solution on collection. A branch of coronary arteries was cannulated for perfusion. The remaining arteries were ligated to prevent leaking of the perfusate. Tyrode solution, equilibrated with 95% O_2_ and 5% CO_2_, was then perfused for 10 minutes. The components of the Tyrode solution include (in mmol/L): NaCl, 140; KCl, 5.4; MgCl_2_, 1.2; NaH_2_PO_4_, 0.33; CaCl_2_, 1.8; glucose, 10; and HEPES, 5 (pH 7.4 with NaOH). After 10‐minute perfusion, digestion buffer was perfused for 15 minutes. The digestion buffer contained (in mmol/L, except described otherwise): NaCl, 125; KCl, 4.75; MgSO_4_, 1.18; KH_2_PO_4_, 1.2; EGTA, 0.02; glucose, 10; taurine, 58; and creatine, 25, in addition to BSA 1%, MEM amino acid 2%, MEM nonessential amino acid 1%, and MEM vitamin solution 1% (Invitrogen). The tissues were then digested with the same digestion buffer containing 150 U/mL type II collagenase (Worthington) for 15 to 20 minutes. After digestion, digestion buffer without collagenase was perfused to wash tissues for 5 minutes. All enzymatic isolation procedures were performed at 37°C, and the perfusion pressure was maintained at 70 to 90 cm H_2_O. The tissues from aortic stenosis patients were directly subjected to cutting and digestion without prior coronary perfusion. The success rate of isolating cells suitable for patch‐clamp experiments was ≈70%, and the cell viability was 30% to 70% depending on tissue condition. All chemicals were purchased from Sigma unless otherwise stated.

### Whole‐Cell Patch‐Clamp Studies and Action Potential Recording

Patch‐clamp experiments were performed as previously described.^12^ Briefly, all experiments were performed at 36°C of chamber temperature, which was regulated by a PH‐1 heating platform, SH‐27B solution heater, and TC‐344B temperature controller (Warner Instruments). Axopatch 200B amplifier and pCLAMP‐10 software (Molecular Device/Axon) were used to generate pulse protocols and record data. Pipette electrodes were made from Corning 7056 glass capillaries (Warner Instruments). The pipette resistance was 3 to 5 MΩ in the bath solution. Whole‐cell patch‐clamp techniques were used to record *I*_KAS_. Extracellular NMG (*N*‐methylglucamine) solution contained (in mmol/L): NMG, 140; KCl, 4; MgCl_2_, 1; glucose, 5; and HEPES, 10 (pH 7.4 with HCl). Pipette solution contained (in mmol/L): potassium gluconate, 144; MgCl_2_, 1.15; EGTA, 1; and HEPES, 10 (pH 7.2 with KOH). Various concentrations of calcium chloride were used to generate different concentrations of free Ca^2+^ according to the calculation by Bers et al.^15^ All cells were superfused continuously with the extracellular NMG solution during recording. Current traces were analyzed with Clampfit software (Molecular Device/Axon). Action potentials were recorded with the whole‐cell perforated‐patch technique with the Tyrode solution as extracellular solution and the same pipette solution containing 120 μg/mL amphotericin‐B and 1 μmol/L free calcium. The extracellular solution without and with apamin 100 nmol/L was used to measure *I*_KAS_. All data were corrected for the junction potentials.

### Western Blot Analysis

SK channels have 3 subtypes.^16–17^ Among them, SK2 is the most sensitive to apamin, whereas SK1 and SK3 are insensitive^18^ and moderately sensitive,^19^ respectively. We detected only a small amount of SK3 protein in the human ventricles with a commercial antibody (P0608; Sigma). However, the low signal‐to‐noise ratio on Western blots prevented proper analyses (data not shown). Therefore, we chose to focus our efforts in studying SK2 expression. For each well, 30 μg of homogenate of left atrial and ventricular tissues was loaded on an SDS–polyacrylamide gel and transferred to a nitrocellulose membrane. The blot was probed with the anti‐SK2 polyclonal antibody (1:600, Abcam). Antibody‐binding protein bands were visualized with ^125^I‐protein A and quantified with a Bio‐Rad Personal Fx PhosphorImager. Expression of SK2 for each sample were normalized to glyceraldehyde‐3‐phosphate‐dehydrogenase levels and expressed as arbitrary units (AUs). A right atrial appendage tissue from a 57‐year‐old male patient with paroxysmal atrial fibrillation, who received cardiac surgery, was used as a positive control.

### Immunohistology and Confocal Imaging

Paraffin sections of 4‐μm thickness were incubated at 60°C for 15 minutes. Slides were then deparaffinized in 3 changes of xylene for 5 minutes each and rehydrated through graded ethanol to distilled water. After washing in PBS, the cells were permeabilized in 0.2% Triton X‐100 for 1 hour at room temperature. Then, slides were washed with PBS and blocked with 2% BSA plus 10% normal donkey serum (Jackson ImmunoResearch) for 1.5 hours. The cells were stained with affinity purified goat anti‐human KCNN2 polyclonal antibody (LifeSpan BioSciences) at a concentration of 4 μg/mL overnight. After washing, the cells were incubated with Alexa Fluor 555 labeled donkey anti‐goat IgG (Invitrogen) at 1:200 dilution for 1 hour. Finally, the slides were washed, stained with Hoechst and mounted in VectaShields medium. Samples were examined by scanning laser microscopy (Olympus FV1000) using a ×60 1.42 NA oil immersion objective and a pixel size of 138 nm. Images were obtained by sequential illumination with 405‐, 488‐, and 559‐nm laser light, while fluorescence was collected in the blue (425 to 475 nm), green (500 to 545 nm), and red (575 to 675 nm) ranges. Differential interference contrast images were also collected using the 488‐nm excitation light.

### Statistical Analyses

Comparison of the continuous variables between 2 groups was performed using the Mann–Whitney–Wilcoxon test. Fisher exact test was used to compare categorical variables between the 2 groups. Kruskal–Wallis test was conducted to compare continuous variables among ≥3 groups, with post hoc Mann–Whitney–Wilcoxon test to compare differences between any 2 groups. Comparison of APD in the absence and presence of 100 nmol/L apamin was performed using Wilcoxon signed rank test. To compare *K*_d_ of calcium sensitivity of *I*_KAS_ between the HF and the non‐HF groups, extra sum‐of‐square *F* test was used. All comparisons were performed to test 2‐tailed methods and *P*≤0.05 was considered statistically significant. Statistical analyses were performed using SPSS PASW Statistics 17 software (IBM), and Prism 5.0 (GraphPad). Data in the text and figures are presented as median [25th and 75th percentiles] or mean±SEM unless otherwise stated.

## Results

### Clinical Characteristics

The left ventricular ejection fraction was significantly lower in the HF group (19% [15% and 22%]) than in the non‐HF group (55% [51% and 64%], *P*<0.001). Nine (75%) patients in the HF group and none in the non‐HF group had a history of ventricular arrhythmias. In the HF group, 9 (75%) patients had an implantable cardioverter‐defibrillator and 9 (75%) had a left ventricular assist device (VAD) as a bridge to orthotopic heart transplantation. The 9 patients with a VAD waited for 208.5 days [80.75 and 319.5 days] before receiving a heart transplant. Table 1 summarizes the clinical characteristics. All 12 HF group and 11 non‐HF group patients were included in the single‐cell studies.

**Table 1.** Clinical Characteristics

	HF (n=12)	Non‐HF (n=11)	*P* Value
Age, y	45 [38; 53]	85 [77; 88]	<0.001
Male gender, %	11 (92)	5 (45)	0.027
CAD, %	4 (33)	1 (9)	0.317
LVEF, %	19 [15; 22]	55 [51; 64]	<0.001
Clinical VT/VF, %	9 (75)	0 (0)	<0.001
ICD, %	9 (75)	0 (0)	<0.001
VAD, %	9 (75)	0 (0)	<0.001
ACEI or ARB, %	6 (50)	3 (27)	0.400
β‐Blocker, %	5 (42)	6 (54)	0.684
Antiarrhythmic agents, %	5 (42)	1 (9)	0.155
Inotropic agents, %	4 (33)	0 (0)	0.093

Data were presented as median [25 percentile; 75 percentile] or number (percentage). HF indicates heart failure; CAD, coronary artery disease; LVEF, left ventricular ejection fraction; VT, ventricular tachycardia; device; ICD, implantable cardioverter‐debrillator; VAD, ventricular assist device; ACEI, angiotensin‐converting enzyme inhibitor; ARB, angiotensin receptor blocker.

### *I*_KAS_ in Cardiomyocytes of the HF and Non‐HF Groups

**Figure 1.**
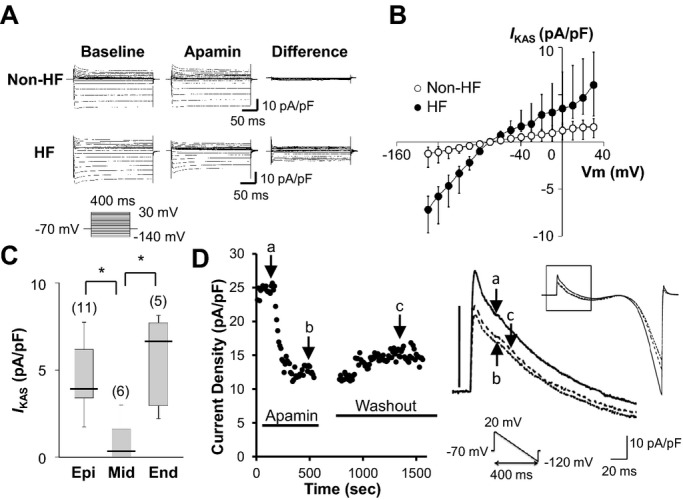
*I*_KAS_ densities of isolated ventricular myocytes. A, Representative K^+^ current traces obtained from a non‐HF (top) and an HF (bottom) ventricular myocyte. The K^+^ currents were recorded with an intrapipette free‐Ca^2+^ of 1 μmol/L in the absence (*I*_K‐baseline_) and the presence of 100 nmol/L apamin (*I*_K‐apamin_). *I*_KAS_ was calculated as the difference between *I*_K‐apamin_ and *I*_K‐baseline_. B, Current‐voltage (I‐V) relationship of *I*_KAS_ obtained from failing ventricles (n=22 cells from 11 patients) and nonfailing ventricles (n=16 cells from 8 patients). Current densities were presented as median [25th percentile; 75th percentile]. C, Transmural distribution of *I*_KAS_ in failing ventricles. *I*_KAS_ density at 0 mV with an intrapipette free‐Ca^2+^ of 1 μmol/L recorded from 3 different layers (Epi, epicardium; Mid, midmyocardium; Endo, endocardium). The midmyocardium had lower current density than the other 2 layers (*P*=0.005 for Kruskal–Wallis test; *P<0.05 for post hoc Mann–Whitney–Wilcoxon test). D, The graph shows the time course of total K^+^ currents measured at 0 mV with ramp‐pulse protocol (test pulse: between +20 and −140 mV; holding membrane potential: −80 mV; pulse frequency: every 6 seconds) in a cardiomyocyte isolated from a failing heart. The current density during apamin (b) and after washout (c) showed only slight differences, indicating incomplete washout of apamin. HF indicates heart failure.

Characteristics of *I*_KAS_ were studied in the ventricular myocytes isolated from HF and non‐HF hearts using patch‐clamp techniques. Figure 1A shows representative whole‐cell K^+^ current traces recorded in the absence and presence of 100 nmol/L apamin with an intrapipette free Ca^2+^ concentration of 1 μmol/L. *I*_KAS_ were detected as 100 nmol/L apamin‐sensitive K^+^ currents (Figure 1A, right panels). A total of 22 cells from 11 hearts from the HF group and 16 cells from 8 hearts from the non‐HF group were studied. Myocytes from the HF group were found to have significantly higher *I*_KAS_ values compared with cells from the non‐HF group. Current‐voltage relationship of *I*_KAS_ demonstrates that both inward and outward *I*_KAS_ were increased in cells from the HF group (Figure 1B). *I*_KAS_ values measured at 0 mV were 4.22 [2.86 and 6.96] pA/pF for myocytes from the HF group (n=11 patients) and 0.98 [0.54 and 1.72] pA/pF for myocytes from the non‐HF group (n=8 patients, *P*=0.008).

To study transmural distributions of *I*_KAS_, cells were isolated from 3 different layers (endocardial, mid, and epicardial layers). *I*_KAS_ were significantly lower in the cardiomyocytes from the mid layer of the HF group than those from the epicardial and the endocardial myocytes (Figure 1C, *P*=0.005 for Kruskal–Wallis test, *P*<0.05 for post hoc Mann–Whitney–Wilcoxon test between the midmyocardial layer versus the epicardial or the endocardial layers).

Figure 1D shows time course of recovery of *I*_K_ from inhibition with apamin (100 nmol/L) obtained in a failing ventricular myocyte. *I*_K_ were activated with 1 μmol/L intrapipette Ca^2+^ and repetitive ramp‐pulses. *I*_K_ was rapidly inhibited with an application of apamin (100 nmol/L). However, *I*_K_ was only partially reversible on washout.

### Steady‐State Ca^2+^ Sensitivity of *I*_KAS_ in HF and Non‐HF Cardiomyocytes

**Figure 2.**
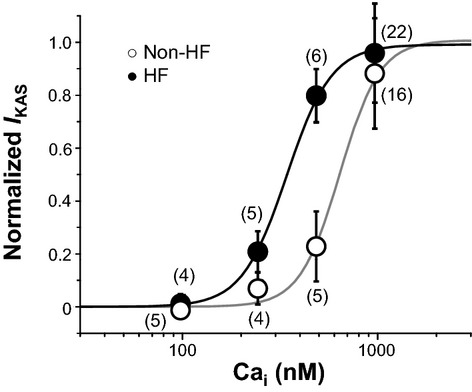
Steady‐state Ca^2+^ dependency of *I*_KAS_ in non‐HF and HF ventricular myocytes. *I*_KAS_ was normalized to the maximal *I*_KAS_ with a free Ca^2+^ of 10 μmol/L and plotted as a function of Ca^2+^ concentration. The data were fitted with Hill equation: *y*=1/(1+[*K*_d_/*x*]^*n*^), where *y* indicates the normalized *I*_KAS_ and *x* is the intrapipette free calcium; *K*_d_ is the concentration of intrapipette free calcium at half‐maximal activation of *I*_KAS_; and *n* is the Hill coefficient. Error bars represent SEM. Numbers in parentheses indicate the number of cells patched. Normalized currents were presented as mean±SEM. HF indicates heart failure.

To further elucidate the underlying mechanisms of the *I*_KAS_ upregulation in HF myocytes, a steady‐state Ca^2+^ sensitivity of *I*_KAS_ was measured using various intrapipette Ca^2+^ concentrations. *I*_KAS_ was normalized by the maximal currents and plotted against Ca_i_. The data were fitted with the Hill equation, yielding significantly lower *K*_d_ values for the HF than for the non‐HF group (HF: 345±4 nmol/L versus non‐HF: 605±28 nmol/L). Hill coefficients were not significantly different between 2 groups (HF: 3.14±0.11 versus non‐HF: 3.79±0.61). As shown in Figure 2, the curve of Ca^2+^‐dependent *I*_KAS_ was left‐shifted in the HF myocytes compared with the non‐HF myocytes (*P*<0.001). Each cell was exposed to only one Ca_i_ concentration. Therefore, the number in Figure 2 is equal to the number of cells studied.

### Measurement of Action Potential in HF and Non‐HF Cardiomyocytes

**Figure 3.**
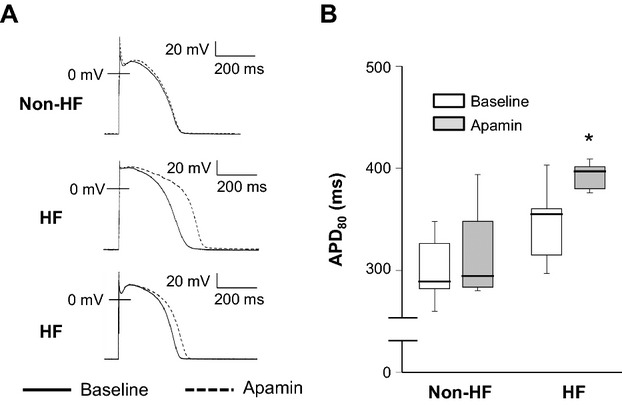
Effects of apamin on APD in ventricular cardiomyocytes. A, Action potentials recorded in 1 non‐HF and 2 HF ventricular myocytes at baseline (solid line) and in the presence of 100 nmol/L apamin (dotted line). B, The box plots depict APD_80_ in various conditions (*P<0.05 for Mann–Whitney–Wilcoxon test). APD_80_ were presented as median [25th percentile; 75th percentile]. HF indicates heart failure.

Next, effects of apamin on APD_80_ were studied using a perforated‐patch method. Although apamin (100 nmol/L) had only small effects on APD_80_ in the non‐HF myocytes, it significantly prolonged the APD_80_ in the HF myocytes by 11.8% (Figure 3). These results indicate that *I*_KAS_ is important in maintaining the repolarization reserve in failing ventricles.

### Western Blot Analysis

**Figure 4.**
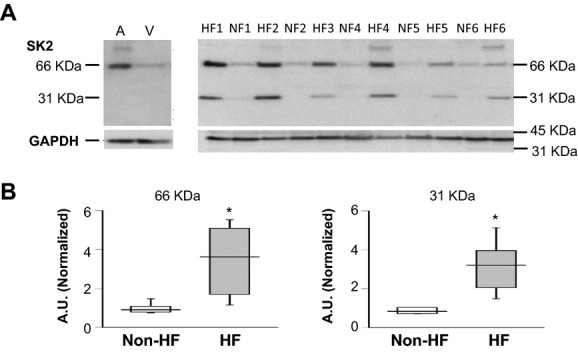
SK2 proteins in HF and non‐HF (NF) ventricles. A, Left, Western blot analysis of atrial and ventricular tissues from a 57‐year‐old male patient with paroxysmal atrial fibrillation and normal LVEF. Right, Western blot analysis of SK2 and glyceraldehyde‐3‐phosphate‐dehydrogenase (GAPDH) in non‐HF (n=5) and HF (n=6) human ventricles. B, Aggregated results of SK2 protein (66 kDa) and presumed SK2 short form protein (31 kDa) expression in non‐HF and HF groups. The signal intensity was normalized to the GAPDH. Data are presented as median [25th percentile; 75th percentile]. HF indicates heart failure; LVEF, left ventricular ejection fraction.

Because a large portion of *I*_KAS_ is carried by SK2 channels,^20^ we examined expression levels of SK2 protein in the tissue homogenate. Using a commercial antibody raised against an immunogen peptide located between residues 450 to 560 of human SK2, we confirmed the previous observations that SK2 channels were expressed abundantly in nonfailing atria but weakly in nonfailing ventricles^21^ (Figure 4A, left, 66‐kDa bands). However, the failing ventricles had significantly higher expression of SK2 protein than the nonfailing ventricles (Figure 4A, right). Protein bands with molecular mass of 30 kDa were also significantly increased in the failing ventricles (Figure 4A, right). The SK3 is detected at low levels. The signal‐to‐noise ratio of SK3 was too low to accurately analyze the SK3 protein concentration. The low SK3 protein expression is consistent with that found by other investigators.^19^

### Immunohistochemical Staining Study

**Figure 5.**
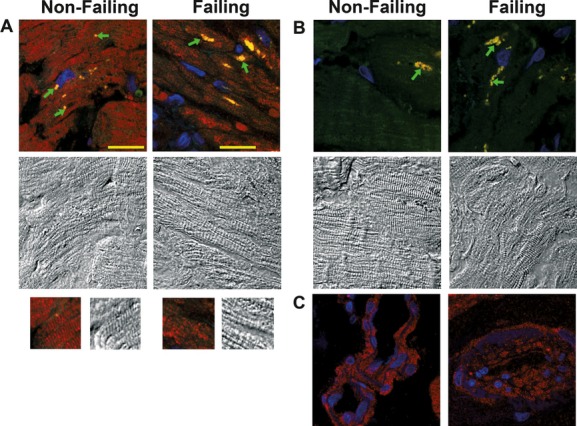
Anti‐KCNN2 immune reactivity in human cardiac tissue. A, Color and black‐and‐white panels show confocal fluorescence images and differential interference contrast (DIC) images, respectively, taken from nonfailing and failing hearts stained for KCNN2. Arrows denote areas of yellow fluorescence that were also detectable in the absence of the secondary antibody and result from the overlap of unspecific red and green autofluorescence in the tissue from nonfailing and failing hearts. B, Confocal fluorescence and DIC images taken from sections that were incubated with the secondary antibody only. C, Anti‐KCNN2 immunofluorescence in small‐artery walls. Scale bar, 20 μm.

We performed immunofluorescence confocal imaging to determine the subcellular distribution of SK2 channel proteins. Representative examples from a nonfailing and a failing heart are shown in Figure 5. In non‐HF cardiomyocytes, anti‐SK2 immune reactivity (red signal; blue, nuclei) displayed a regular striated appearance, compatible with SK2 channel clusters being distributed along transverse‐tubular membranes (see also higher‐magnification views at the bottom). Spatial distribution of SK2 protein became more disorganized in cardiomyocytes from failing hearts, suggesting partial loss of SK2 channel clusters at striations. Both HF and non‐HF cardiomyocytes exhibited areas of unspecific yellow fluorescence, which were also present in the absence of the fluorescent secondary antibody and therefore result from the overlap of unspecific red and green autofluorescence in processed tissue. Small arteries in both nonfailing and failing hearts were found to express SK2, in agreement with previous observations by others.^22^

## Discussion

In this study, we report several novel findings: (1) *I*_KAS_ was upregulated in failing human ventricles; (2) the distribution of *I*_KAS_ was transmurally heterogeneous with the midmyocardial layer expressing significantly less *I*_KAS_ than the epicardial and the endocardial layers; (3) the upregulated *I*_KAS_ significantly contributes to the repolarization of failing ventricular myocytes; (4) both increased SK2 protein and increased Ca^2+^ sensitivity underlie the upregulation of *I*_KAS_; and (5) partial loss of SK2 channel clusters at striations in failing ventricles.

### *I*_KAS_ and Ventricular Arrhythmogenesis in HF

Abnormal Ca_i_ handling is one of the major underlying mechanisms of arrhythmias associated with HF. For example, diastolic Ca_i_ can be elevated in HF due to the increased Ca^2+^ leak from hyperphosphorylated ryanodine receptors^23–24^ and reduced Ca^2+^ uptake by sarcoplasmic reticulum Ca^2+^‐ATPase.^24^ The increased Ca_i_ can activate the Na^+^‐Ca^2+^ exchanger, which tends to prolong APDs, resulting in EADs and triggered activity.^25^ The APD‐prolonging effects of Ca_i_ accumulation are counterbalanced by the activation of various K^+^ currents in normal ventricular myocytes. However, because of the downregulation of multiple major K^+^ currents (*I*_to_, *I*_Ks_, *I*_Kr_, *I*_K1_, and *I*_KATP_),^3–6^ the HF cardiomyocytes have reduced ability to shorten the APD. This reduced repolarization reserve may play an important role in proarrhythmia in failing ventricles. In our study, some of the midmyocardial layer myocytes in failing ventricles did not show increased *I*_KAS_. These cardiomyocytes might be particularly vulnerable to afterdepolarizations and triggered activities.

While the upregulation of *I*_KAS_ may be antiarrhythmic, it can also be proarrhythmic. We showed that *I*_KAS_ is upregulated in epicardial and endocardial myocytes in failing human ventricles. In these cells, *I*_KAS_ is almost constitutively active due to the increased Ca^2+^ sensitivity. Although Ca_i_ in the range of 300 to 400 nmol/L activates only 10% of maximal *I*_KAS_ in nonfailing myocytes, 50% of maximal *I*_KAS_ can be activated in failing myocytes (Figure 3). The activation of *I*_KAS_ can lead to excessive shortening of APDs in these cells during increased Ca_i_ states (such as during VF or during sustained ventricular tachycardia), which can promote late phase 3 EAD and triggered activities.^12,23^ Because elevated Ca_i_ may persist after successful defibrillation,^11,26^ the increased Ca_i_ can activate *I*_KAS_, leading to further shortening of APDs and spontaneously recurrent VF (electrical storm).^8,12^ Therefore, the upregulation of *I*_KAS_, by itself, can be one of the important underlying mechanisms of “VF begets VF.” In addition, due to the heterogeneous distributions of *I*_KAS_, an increase in Ca_i_ caused by tachycardia or sympathetic activation may cause different magnitudes of *I*_KAS_ activation and thereby can increase transmural heterogeneity of APDs, which is known to be proarrhythmic. Taking these facts together, it is reasonable to speculate that the upregulation of *I*_KAS_ can be both antiarrhythmic and proarrhythmic in HF.

### Increased SK2 Proteins in Failing Ventricles

Macroscopic whole‐cell currents are determined by expression levels of channel proteins and ion channel functions such as channel open probability. The upregulation of *I*_KAS_ can be achieved by an alteration of the expression of SK channels and/or the Ca^2+^‐sensitivity of *I*_KAS_. It has been reported that 3 different subtypes of SK channels (SK1, SK2, and SK3) are expressed in human hearts.^27^ Among these channel subtypes, SK2 is known to be the most sensitive to apamin and carry most of the *I*_KAS_.^20^ Our Western blot analyses using anti‐SK2 channel specific antibody showed that bands with a molecular mass of 66 kDa, corresponding to the known molecular size of SK2 variant 1 (NM_021614), were increased by 3‐fold in the failing ventricles compared with the nonfailing ventricles. Because we detected a similar band in the human atria, which is known to express SK2 channels, we believe that SK2 channels were overexpressed in the failing ventricles of these patients. All these findings are consistent with our hypothesis that increased SK2 channel protein expression gave rise to increased *I*_KAS_ in failing ventricles. An intriguing finding is the appearance of protein bands at a molecular mass of 31 kDa in the failing ventricles but little or none in the nonfailing ones. Although the true identity of these smaller bands was unclear, a known isoform of human SK2, SK2‐s (NM_170775.1), might contribute to these 31‐kDa bands. There are no previous reports on the expression and function of SK2‐s in failing human hearts. Identification and characterization of this protein will be informative for further understanding the biophysical properties of *I*_KAS_ in failing ventricles.

### Subcellular Distribution of SK2 Protein

The subcellular distribution of SK2 protein in human ventricular myocytes is unknown. Previous immunohistochemical analyses have demonstrated that SK2 proteins are localized along the Z‐line in human and canine ventricular myocytes.^14,17,28^ Our immunofluorescence confocal imaging revealed SK2 protein localization along the Z‐line in nonfailing heart that is similar to that seen in murine atrial myocytes. On the contrary, in failing hearts, the distribution of SK2 proteins was more diffuse, compatible with the notion that the mechanism responsible for SK2 protein alignment in physiological conditions is disrupted in failing ventricular cardiomyocytes. Possible reasons include structural remodeling of the transverse‐axial tubular system^29^ and changes in targeting of the channel protein to or anchoring in the sarcolemma.^30–31^ The functional consequences of these spatial rearrangements will be the subject of future investigations.

### SK2 Proteins and the Upregulation of *I*_KAS_

It has been reported that the gating of rat SK2 channels solely depends on Ca_i_ with a half‐maximal *K*_d_ of 500 nmol/L in *Xenopus* oocytes with an inside‐out patch technique.^32^ A similar *K*_d_ value was obtained in our whole‐cell current measurement in myocytes with intact regulatory proteins of SK channels. Interestingly, the *K*_d_ in the failing ventricular myocytes was significantly lower than the control (314±4 nmol/L versus 605±28 nmol/L). These results suggest that channel open probability is higher in failing ventricular cells than in control cells at the same Ca_i_ (ie, more sensitive to Ca_i_; Figure 2). Recent studies identified several regulatory proteins that coassemble and regulate SK channels.^33–34^ These proteins include calmodulin, protein kinase CK2, and protein phosphatase (PP)2A. The study using a heterologous expression system demonstrated that CK2 can phosphorylate threonine 80 of calmodulin, resulting in a right‐shift of Ca^2+^ sensitivity of SK2 channels. This finding suggests that the left‐shift of Ca^2+^ sensitivity of *I*_KAS_ observed in our study can be caused by a decrease in phosphorylation of calmodulin, by increased CK2 activity and/or increased PP2A activity. Unfortunately, limited availability of fresh human samples prevented us from making any conclusions regarding the role of these regulatory proteins in the current study. According to the study using canine heart failure models by Yeh et al,^24^ the protein expressions of PP1 and PP2A were not altered, but the activity of PP1 was significantly increased in failing atria. Although the role of CK2/PP1 in the regulation of SK channels in cardiomyocytes is not fully understood, there is a possibility that CK2/PP1 can alter the Ca^2+^ sensitivity of *I*_KAS_ in human HF by modulating phosphorylation status of calmodulin, which requires further biochemical studies.

### Study Limitations

There are several significant limitations in this study: (1) our control subjects had aortic stenosis and normal left ventricular ejection fraction because healthy hearts were not available for this study. These control patients are significantly older than the patients with HF, and there is a possibility that aging can reduce *I*_KAS_ in normal human ventricles. However, studies using animal models and normal human samples agreed with the findings that *I*_KAS_ was poorly expressed in the nonfailing ventricles.^14,28^ Therefore, it is reasonable to speculate that *I*_KAS_ expression is not significantly affected solely by age. (2) Some of the patients with HF were taking antiarrhythmic agents. Whether those antiarrhythmic agents could be washed out during the cell isolation and patch‐clamp experiments remains unknown. It is possible that some of the antiarrhythmic agents may affect the function and expression of SK2 channels. (3) Because the number of patients was small and the study design was cross‐sectional (ie, done at the time of transplant), we cannot determine whether the upregulation of *I*_KAS_ can alter the patients' mortality and incidence of arrhythmias. (4) Because the time window for the experiments was limited to no more than 10 hours after isolation, we were not able to study a large number of cells in each patient. As indicated in a recent study using human left ventricular wedge preparation,^35^ the distribution of M cells can be highly heterogeneous in human ventricles. Therefore, it is possible that we have studied both M cells and non‐M cells in the midmyocardial layer, leading to a large variation of the current densities.

## Conclusions

Our study for the first time demonstrated the heterogeneous upregulation of *I*_KAS_ in failing human ventricles. Both increased protein expression and increased Ca^2+^ sensitivity can contribute to the *I*_KAS_ upregulation. Because all known K^+^ currents are downregulated in HF, the *I*_KAS_ plays a unique role in maintaining repolarization reserve in failing human ventricles. We propose that *I*_KAS_ plays an important role in ventricular repolarization in failing human ventricles and thereby pose as a new therapeutic target in patients with HF.
